# Redefining robotic image-guidance – tomographic visualization of lesions during prostate cancer surgery via gantry-free robotic SPECT

**DOI:** 10.1007/s00259-026-07987-z

**Published:** 2026-07-01

**Authors:** A. C. Berrens, K. Pirkovets, S. Azargoshasb, L. J. Slof, B. A. Cakal, P. J. van Leeuwen, E. M. K. Wit, M. Sinaasappel, T. Wendler, H. G. van der Poel, M. N. van Oosterom, F. W. B. van Leeuwen

**Affiliations:** 1 Interventional Molecular Imaging Laboratory, Leiden University Medical Center, Leiden, The Netherlands; 2 Department of Urology, Netherlands Cancer Institute-Antoni van Leeuwenhoek Hospital, Amsterdam, The Netherlands; 3 Clinical Computational Medical Imaging Research, Department of Diagnostic and Interventional Radiology and Neuroradiology, University Hospital Augsburg, Augsburg, Germany; 4 Crystal Photonics GmbH, Berlin, Germany; 5 Department of Clinical Physics and Instrumentation, Netherlands Cancer Institute, Amsterdam, The Netherlands; 6 Diagnostic and Interventional Radiology, Faculty of Medicine, University of Augsburg, Augsburg, Germany; 7 Department of Urology, Amsterdam University Medical Center, Amsterdam, The Netherlands

**Keywords:** Robotic surgery, Receptor targeted, Image-guided surgery, Prostate cancer, Interventional nuclear medicine, Augmented reality

## Abstract

**Purpose:**

The introduction of the drop-in gamma probe has advanced intraoperative molecular imaging during prostate cancer surgery. We have been able to convert the sensor’s numeric readout to tomographic images, so-called robotic-SPECT (_Robo_SPECT) and investigate how this is impacted by radiopharmaceutical avidities and drop-in scan metrics.

**Methods:**

The gamma sensor readout was registered with its 3D position and orientation, allowing a custom reconstruction algorithm to generate _Robo_SPECT images. Evaluations occurred in 21 patients; 10 sentinel node procedures (SN; primary prostate cancer) and 11 PSMA-radioguided surgery (recurrent prostate cancer). _Robo_SPECT findings were related to respective pre- and intra-operative controls, including preoperative PSMA-PET/CT and/or SPECT/CT images and fluorescence detection (SN only).

**Results:**

_Robo_SPECT proved to be safe and applicable in a range of conditions. In the SN-group, 26 SN-SPECT/CT lesions were successfully identified with SN-_Robo_SPECT (100%); 3 were tumor positive (sensitivity 100%). Only 73% of SNs were surgically visible with fluorescence imaging. For the PSMA guided group, the 14 lesions identified on PSMA-PET/CT were all visualized with PSMA-_Robo_SPECT (100%); 18 specimens were tumor positive (sensitivity 78% for both PSMA-PET/CT and PSMA-_Robo_SPECT). Preoperative PSMA-SPECT/CT only identified 4 PSMA-lesions (29%). No false positives were seen for _robo_SPECT and all final resection margins were clean. At 6-months 0% of the SN-patients and 20% of PSMA-patients showed biochemical recurrence.

**Conclusion:**

_Robo_SPECT provides 3D context that extends the utility of drop-in gamma tracing and assists the alignment between pre- and intra-operative target perception. Here SN-_Robo_SPECT clearly outperformed fluorescence SN imaging and PSMA-_Robo_SPECT outperformed preoperative PSMA-SPECT/CT imaging.

## Introduction

Robot-assisted laparoscopic surgery is the most clinically applied branch of medical robotics. This forward-looking discipline enables minimally invasive interventions through telerobotic surgeon–patient interactions, relying on a zoomed-in stereoscopic view and finely steerable instruments that enhance surgical dexterity [1, 2]. To support intraoperative decision-making, first-person white-light endoscopic imaging can be enriched with state-of-the-art molecular sensing techniques that offer complementary third-person guidance. The resulting synergies in advances of engineering and molecular imaging enable surgeons to better exploit the robot’s potential to identify and resect diseased tissue [3, 4].

Over the last decade, urological indications have been major drivers of molecular image-guided robotics. These predominantly include pelvic lymph-node metastases in prostate cancer (PCa), addressed through sentinel lymph node (SN)-targeted resections in primary disease [3], and through prostate specific membrane antigen (PSMA)-receptor-targeted lesion dissection in both primary and recurrent PCa [5, 6]. PSMA-targeted procedures may also include resection of local recurrences. All these interventions depend on preoperative imaging that reflects locoregional disease spread, typically through PSMA-positron emission tomography/computed tomography (PET/CT), a direct 3D surgical reference scan for PSMA-targeted approaches.

Before surgery, patients receive a ^99m^Tc-labeled radiopharmaceutical (a gamma emitter with its main energy peak at 140 keV). Tissue avidity is then evaluated using non-invasive nuclear imaging – most commonly single-photon emission computed tomography (SPECT)/CT, the direct reference scan for SN-targeted procedures [7, 8]. During robotic surgery, radiopharmaceutical-avid tissues visualized preoperatively can be traced using a miniaturized tethered drop-in gamma sensor technology [9]. This device not only quantifies emissions (counts/s; cps) but also leverages the six degrees of freedom of the robotic instrument holding it. In both SN- and PSMA-targeted procedures, preoperative signal intensities have been shown to correlate with intraoperative target detection [7, 10]. However, registering static preoperative SPECT/CT images to the deformable soft-tissue environments encountered during surgery remains a considerable challenge [11].

Because the surgical field is highly dynamic, surgeons must continuously adapt their strategy based on scene changes. Molecular precision perception of targets may support such adaptations [4]. Current intraoperative molecular imaging tools often rely on radio- and/or fluorescence-guided techniques [12, 13]. Where the first signals support in-depth guidance, the latter merely tailor superficial target identification [14]. Hybrid tracers – such as indocyanine green (ICG)-[^99m^Tc]Tc-nanocolloid [9] help align both strategies. What hybrid tracers do not influence is how the signals are perceived by the surgical end-user. Despite its superficial character, the ability of near-infrared fluorescence laparoscopy to provide surgeons with a first-person video-view of the target has driven its embodiment in modern robotic platforms [4]. In parallel, the third-person numerical (counts/s) feedback of steerable drop-in gamma sensors can be difficult to interpret. But that has not withheld surgeons from using the technique [3, 15, 16]. A recent consensus paper highlights a preference for image-based, first-person feedback rather than purely numerical, third-person sensor readouts [17]. Thus, underscoring the need for technologies that enable direct visualization of radiopharmaceutical-avid targets within the endoscopic field, for example through augmented-reality approaches.

In open surgery, gantry-free SPECT provides a valuable alternative source of intraoperative tomographic molecular imaging [18, 19], improving the fidelity between preoperative SPECT/CT reference scans and intraoperative findings. Hereby intraoperative tomographic imaging has even been proposed as a replacement for preoperative SN-SPECT/CT [20]. Unfortunately, the external optical-tracking of rigid instruments that is currently used during open surgery [18, 19] cannot be translated to intra-abdominal tracking of a flexible drop-in sensor [21]; Flexible sensors cannot be tracked using external fiducials and require endoscopic video-based solutions [22].

**Fig. 1:**
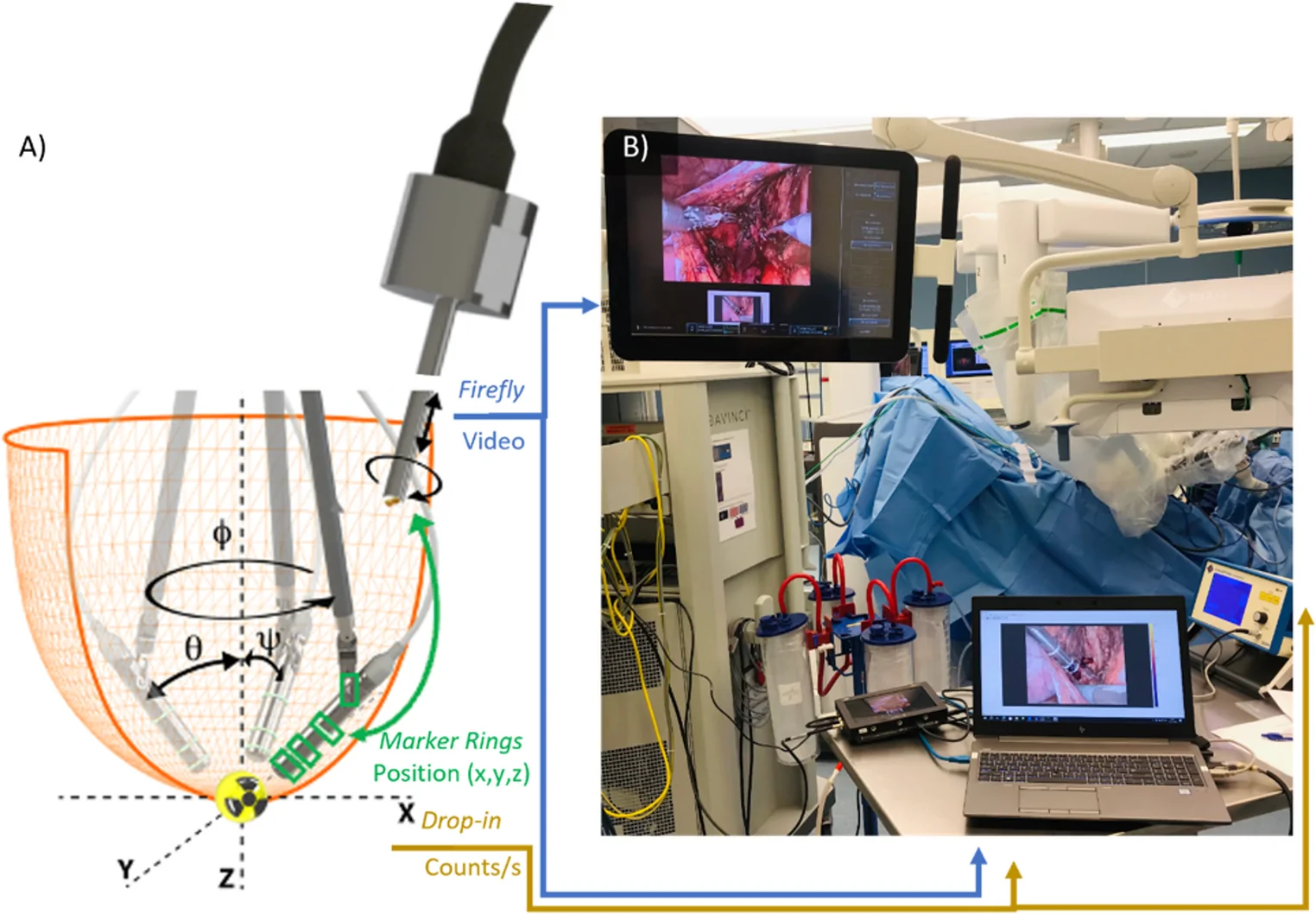
Technical set-up. (**A**) Representation of the drop-in sensor maneuverability in a surgical cavity as monitored using the Firefly Xi endoscope. ϑ represents pitch, Ψ represents jaw, and Φ represents roll; (**B**) Visualization of _Robo_SPECT generation during surgery and augmented reality visualization of the _Robo_SPECT scan on a laptop and in the assistant display via TilePro

Following initial preclinical evaluations [23], a complete redesign of the hardware and software components required for drop-in tracking solutions relying on marker-based video processing was performed (Fig. 1) [22]. This enabled the first preliminary clinical evaluation of da Vinci robot-assisted SPECT (SN-_Robo_SPECT) imaging in four PCa patients [24]. Building on this proof-of-concept, we now:

1. implement improvements in tracking and image-reconstruction pipelines;
2. evaluate the _Robo_SPECT technology (Fig. 1) in a larger clinical cohort of SN patients;
3. benchmark SN-_Robo_SPECT against endoscopic fluorescence SN imaging;
4. expand the _Robo_SPECT application to PSMA-targeted procedures, which convert to lower signal intensities and lower signal-to-background ratios [7]; and.
5. benchmark its detection rate against preoperative SPECT/CT and PET/CT findings.

## Methods

### Patient population

In total, 21 patients with PCa underwent robot-assisted radioguided surgery procedures using ^99m^Technetium (Tc) between 2023 and 2024. Ten patients underwent a routine SN procedure in primary PCa. In the SN group, patients diagnosed with high-risk primary PCa that were scheduled for EBRT were included. All patients had clinical stage cT1c or cT2 tumor, without signs of metastases on PSMA PET/CT within 90 days before surgery according to local protocol ([^68^Ga]Ga-PSMA-11, [18 F]DCFPyL or [18 F]PSMA-JK-7; Table 1). Eleven patients with recurrent PCa underwent salvage PSMA-targeted surgery with additional unilateral lymph node dissection as part of clinicaltrials.gov NCT03857113 (institutional review board (IRB) approved study extension IRBd-22–231). The patients included in the PSMA-targeted surgery group (salvage setting) were selected based on a PSA of ≤ 4 ng/ml, a maximum of 3 soft-tissue lesions on PSMA PET/CT ([^68^Ga]Ga-PSMA-11, [^18^F]DCFPyL or [^18^F]PSMA-JK-7) performed within 90 days before surgery and suitable for salvage surgery according to NCT03857113.

**Table 1:** Patient characteristics and preoperative diagnostic data

Parameter	Sentinel Node (*n* = 10)	PSMA-Targeted (*n* = 10^#^)
Preoperative data		
Age (yr), median (IQR)	64 (61–69)	71 (66–75)
initial prostate-specific antigen (ng/ml), median (IQR)	11 (8.3–17.5)	1 (0.4–2.0; prior to salvage surgery)
LNI risk (%), median (IQR)	18 (14–30)	n/a
Clinical T stage, n (%)		
cT1c	6 (60)	n/a
cT2	4 (40)	n/a
Radiological T stage, n (%)		
mT2	7 (70)	n/a
mT2b	1 (10)	n/a
mT2c	1 (10)	n/a
mT3b	1 (10)	n/a
Staging according to PSMA PET/CT, n (%)	9 (90)*	10 (100)
Lesions identified at preoperative PET/CT, n (%)		
0	10 (100)	0 (0)
1	0 (0)	7 (70)
2	0 (0)	2 (20)
3	0 (0)	1 (10)
Time between PET/CT and surgery (days), median (IQR)	98 (57–176)	48 (33–65)

^#^One patient was excluded due to the lack of surgical data. *One patient only received MRI. *IQR* interquartile range, *LN* lymph node, *LNI* Lymph node involvement, *MBq* Megabecquerel, *PET* positron emission tomography, *PSMA* prostate-specific membrane antigen, *SN* sentinel lymph node, *SPECT* single-photon emission computed tomography

### Preoperative imaging

The SN patients were injected with the radioactive and fluorescent ICG-[^99m^Tc]Tc-nanocolloid (GE Healthcare, Eindhoven, The Netherlands; ~222MBq; ~7 h prior to surgery), as described before [7, 9]. Early and late lymphoscintigrams (at 15 min and 2 h after injection) were followed by a SPECT and low-dose CT scan. The preoperative SPECT/CT was used to define the general anatomical location of sentinel nodes (SNs).

The patients included in the PSMA-targeted surgery group received an intravenous injection of [^99m^Tc]Tc-PSMA I&S (~ 566MBq; ~21 h prior to surgery). Preoperative SPECT/CT was performed at the morning of surgery to assist in surgical planning.

### Sensor design and design tracking fiducials

**Fig. 2:**
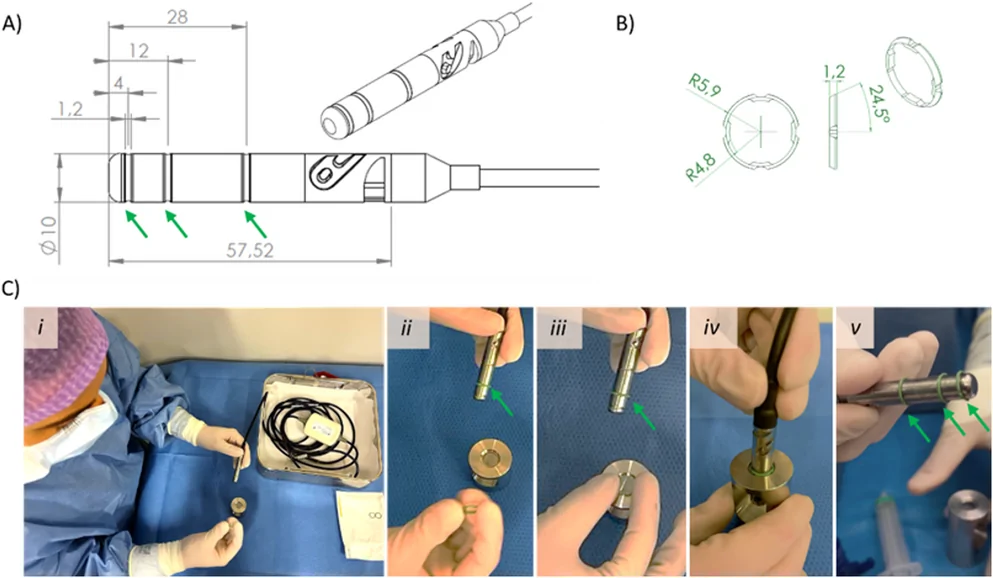
Hardware required for marker-based drop-in sensor tracking. (**A**) Sterilizable and CE-marked drop-in gamma sensor designed with three circular engravings, so-called “notches” in the titanium outer housing. (**B**) PEEK marker rings that are custom made to fit into the “notches” and that have a 25^o^ angled facing edge towards the cable end of the sensor. (**C**) In a sterile surgical environment, the marker rings can be reproducibly attached by a bedside assistant using a custom-made mounting tool. Green arrows indicate the notches and/or PEEK marker rings located therein

The titanium housing of the medical CE-marked second-generation drop-in sensor (Crystal Photonics GmbH, Berlin, Germany) includes three circular engravings (Fig. 2A). Marker-based tracking was facilitated by using, disposable, clinical-grade high-contrast green PEEK (Biocompatible PEEK Medical Grade - TECAPEEK MT Green, Ensinger GmbH, Nufringen, Germany). This material is tested according to standard ISO 10,993 and can be used with either steam or plasma sterilization. The rings (Fig. 2) were produced with a 3 axis CNC turning machine with live tooling (DMG CTX 310, DMG MORI AG, Bielefeld, Germany) and were designed in SolidWorks (Dassault Systèmes SolidWorks Corp., Vélizy-Villacoublay, France) to be press-fitted onto the three notches within the housing of gamma sensor (Fig. 2C). With the PEEK rings attached the sensor remains compatible with a standard 12 mm trocar (Fig. 1; final dimension 11.8 mm).

To allow easy and controlled attachment of the rings while wearing gloves, we fabricated a mounting tool out of sterilizable stainless steel 316 (see Fig. 2C). The oblique ring-design prevents that the functionalized drop-in sensor snags on tissue or the trocar when it is retracted from the patient by the bedside assistant.

### Surgical procedures

All procedures were performed using a multiport Da Vinci Xi surgical system (Intuitive Surgical Inc., Sunnyvale, CA, USA). SN procedures benefitted from the system’s integrated Firefly fluorescence camera’s ability to detect ICG-[^99m^Tc]Tc-nanocolloid. For all procedures a shock-resistant, sterilizable second-generation drop-in gamma sensor was used (Crystal Photonics GmbH, Berlin, Germany) [5] (Fig. 2). The ring-functionalized drop-in gamma sensor was inserted into the abdominal cavity either through or next to a 12-mm trocar within the Alexis portal system (Applied Medical Corp., Rancho Santa Margarita, CA, US). The operating surgeon was able to independently grasp and maneuver the drop-in gamma sensor using the ProGrasp Forceps (Fig. 1). Via the Da Vinci TilePro, the input of the sensory readout was directly displayed in the surgeon’s view as counts/s (cps). Signal-to-background ratios (SBRs) were recorded; the maximum sensor counts before final resection of the lesion (SN or PSMA-targeted node) were considered the signal value and the average counts on the direct surrounding tissue (< 2 cm away from the target) were considered background value. All data needed for the _Robo_SPECT images were acquired by simultaneously recording counts and probe trajectories while the surgeon scanned the surgical targets, only providing a minor delay on the surgical care (see below). However, to minimize the impact of the research evaluation on patient care, _Robo_SPECT reconstructions were only used for confirmation of the resected target.

At the end of the procedure, all the tissue was remeasured ex vivo using the same drop-in gamma sensor. Lesions were classified as being ‘hot’ according to surgeon’s interpretation during the procedure. The median procedure time was 76 min for SN and 135 min for PSMA.

### Tracking software

The output from one “eye” of the Firefly endoscope, recorded with a Blackmagic HyperDeck Studio HD Plus (Blackmagic Design Pty Ltd, South Melbourne, Australia) at a resolution of 1920 × 1080, was used to capture the surgical procedure. Custom computer-vision based algorithms (C++; OpenCV) that consider both color and geometry of the 3 PEEK rings supported vision-based tracking of both position and orientation (5 degrees-of-freedom) [22]. Following a checkerboard calibration of the endoscope’s intrinsic and extrinsic camera parameters [21, 25], the known geometric arrangement of the markers (an asymmetric 3-ring pattern [22]) enabled determination of the sensor tip’s pose in 5 degrees of freedom (x, y, z position, yaw and pitch angle). The resulting 3D movement trajectory was digitized using custom algorithms (MATLAB^®^, the MathWorks, Inc., Natick, MA, United States).

To overcome gaps in the tracking due to e.g. 0^o^ angles with respect to the camera, dirty lens, or overlapping tissue, we employed track “stitching” (for lost frames) and track filtering (for tracking outliers). As the tracked sensor position is directly related to the vision at the endoscope tip, operating surgeons were asked to fix the laparoscope position while scanning the target.

### SPECT via look-up table (LUT)

Our initial work [24] relied on the solid-angle principle for the 3D reconstruction of the radioactivity distribution. Although the solid-angle principle offers reasonable and practical SPECT reconstructions, studies show that employing Monte Carlo based look-up tables provide faster and more accurate system modelling to be used in the MLEM algorithm for SPECT [18, 26, 27]. A set of previously acquired measurements of a point source is used to model the contribution of each voxel to a measurement. This involved modeling the gamma sensor (based on geometry and materials of collimator, shielding and crystal) in the Monte-Carlo simulation GATE (OpenGATE, 2020) including scatter and attenuation in all modelled components and defining a point source emitting gamma rays with a monoenergetic energy of 140 keV (the photopeak of ^99m^Tc) [27]). This results in a far more realistic forward model for the reconstruction in comparison to geometric or analytical ones. The lookup table covered a range of −20 mm to + 20 mm (with a step size of 2 mm) in the sensor-right and sensor-left directions, and from 8 mm to 67 mm (with a step size of 1 mm) in the probe forward-direction, which yields to a total of 26,460 simulations. The material between point source and probe was assumed to be air, and as such, attenuation and scatter in tissue were not considered. For each simulation, around 2,500,000 gamma emissions are simulated, based on previous experiments and the fact that the probe’s geometry is far simpler than a typical SPECT detector/collimator setup. With the applied energy window of 120–160 keV, the number of detected counts among the total number of emitted rays leads to the detection probability p(k) = P[detected | emitted from relative voxel position k], where k = 1:K is the number of voxel positions in the probe-relative LUT grid, as described before [28]. Following the recommendation of [27], we model dead time in the electronics and the most relevant physical interactions of gamma rays and collimator/crystal (photoelectric effect, Compton and Rayleigh scattering, Bremsstrahlung, electron ionization). Aspects that were excluded in the simulation where characteristic X-ray emissions from the crystal, afterglow/phosphorescence, non-linear responses of the crystal, potential intrinsic radioactivity of the crystal due to contamination, backscattering from the electronics, photon absorption by the crystal encapsulating material, thermal drift of SiPM, dark counts, and other losses in the interface between crystal and SiPM.

### _Robo_SPECT reconstruction

Custom tomographic reconstruction algorithms (MATLAB, the MathWorks, Inc., Natick, MA, USA) were used to correlate the sensor readout, its respective position and orientation, and the LUT.

**Fig. 3:**
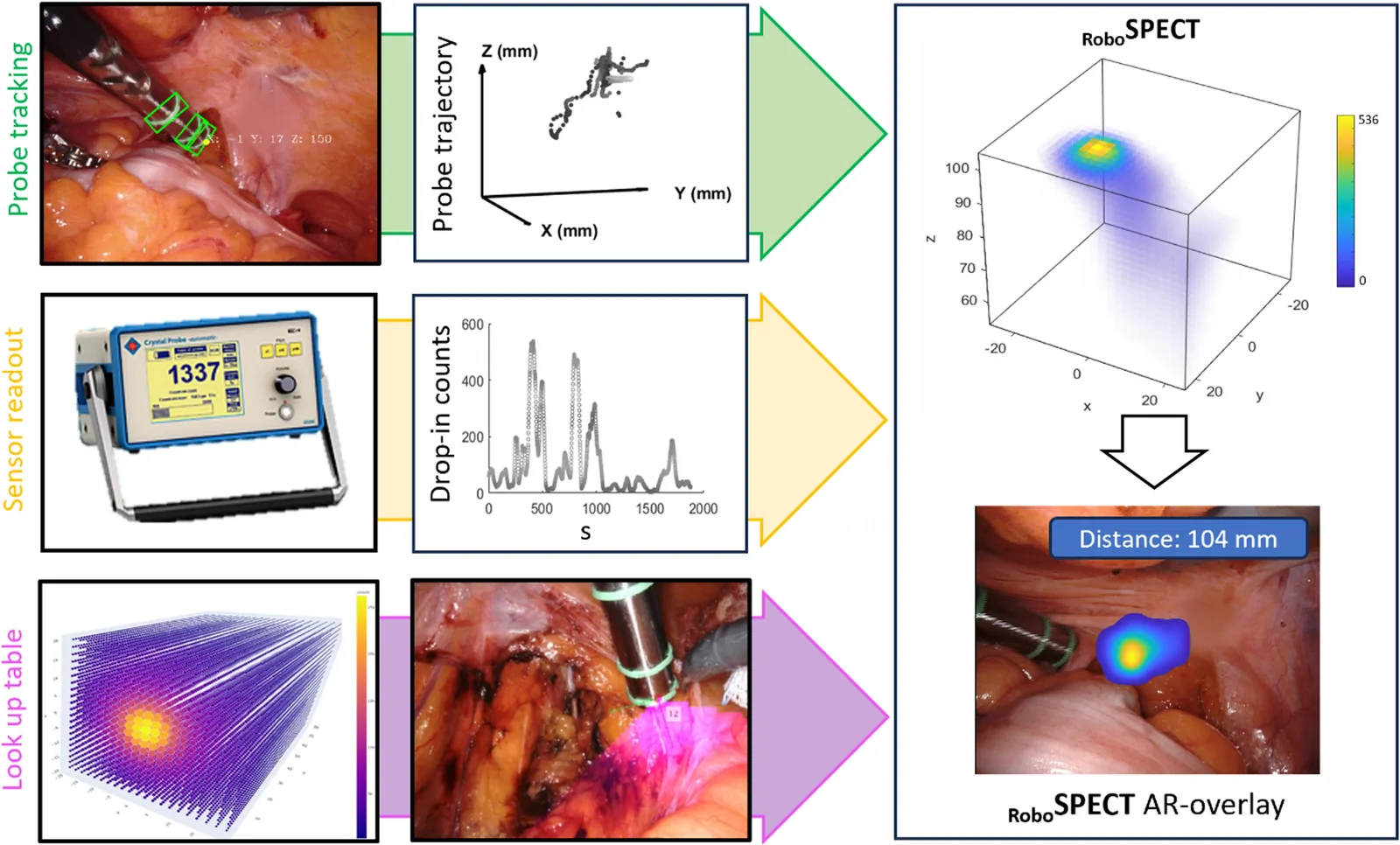
Multiplexing of data streams to enable _Robo_SPECT generation. Starting with an endoscopic video wherein the 3 green PEEK marker rings help elucidate the scan path (x, y, z position; yaw and pitch angles over time) of the drop-in sensor (workflow in the green arrow) was synchronized with the recording of the drop-in sensor readout (counts/s) over time (workflow in the yellow arrow). Via a specific lookup table (LUT) the drop-in sensor view was estimated at different positions (workflow in the pink arrow). A custom maximum likelihood expectation maximization (MLEM) reconstruction algorithm converted the synchronized data inputs into a tomographic _Robo_SPECT image (top right image) that could be overlayed on the endoscopic view using augmented reality (AR; bottom right image) whereby the distance of the endoscope tip to the target is presented

The above-mentioned video-tracking allows defining of the position (x, y and z) and orientation (yaw (Ψ) and pitch (ϑ)) with respect to the ^99m^Tc-avid targets (Fig. 1). Given the mismatch in data density (i.e., 30 Hz video tracking and 10 Hz gamma sensing), data synchronization and interpolation was required to register the sensor readout to the spatial information (Fig. 3). These synchronized readouts were complemented by a drop-in look-up table (LUT; 40 × 40 × 59 mm volume: Fig. 3) that was generated using GATE Monte Carlo simulations and that considers specific sensor characteristics (collimator detection window radius, length and material, detector shape and material, shielding around detector) [27]. The resulting LUT was validated with the product specifications (forward facing conical detection window of 45^o^; collimated shielding of 99% at 140 keV; local resolution < 1 cm; sensitivity > 17000 cps/MBq). The combined data inputs were coupled with a calculation of a system matrix for a subsequent image reconstruction.

Combined this information resulted in a scan-specific system matrix consisting of one row per reading and one column per voxel to be reconstructed. For each acquisition i, the system matrix element H(i, j) is populated by transforming reconstruction voxel j (defined in world coordinates, j = 1:N) into the probe-relative coordinate frame using the tracked probe pose at readout i, and retrieving the corresponding LUT entry p(k). An iterative MLEM reconstruction [18, 26, 27, 29] was applied in a 100 × 100 × 100 mm^3^ volume with 2 mm isotropic voxels placed in front of the sensor at the beginning of the scan. The MLEM formulation did not include any form of regulation or noise suppression. During iterative image refinement, information from multiple angles and positions was incorporated to compensate for the projective nature of the acquisition ultimately yielding high-resolution volumetric reconstructions. An early reconstruction stop was based on a threshold for solving the relative change in the Frobenius norm with 100 iterations allowed as maximum. To avoid artifacts at the boundaries of the _Robo_SPECT scans, readings within a 15 mm vicinity of the boundaries were disregarded during the reconstruction. Average scan times were 40 s for one target (IQR: 19–48 s). The reconstruction process took approximately 3 min per scan and could be ran in parallel to ongoing surgical resections (see Fig. 1).

Using an automatic 2D augmented reality projection, the 3D _Robo_SPECT scans were projected onto the endoscopic white-light view (filtered with a 1.5 cm-radius Gaussian filter; Fig. 3). Hereby, the distance between the target (i.e., its weighted center of gravity) and the laparoscope’s tip could be depicted (Fig. 3).

### Complications

Use of the ring-functionalized drop-in sensor was safe and did not instigate surgical complications. Postoperative complications were observed in 3 PSMA patients (constipation – clavien-dindo 1; urinary retention, lymphocele – clavien-dindo 2; urinary retention. Transurethral catheter longer in situ – clavien-dindo 1). There was no relevant blood loss for any patients.

### Histopathology

SNs were fixed in formalin, cut into 2 mm segments, embedded in paraffin, and stained with hematoxylin and eosin (HE) and the middle slide was additionally stained with a pancytokeratin staining. Non-SNs were cut in 3-mm slices, usually longitudinal to the long axis, and only one HE slide per block was made. During PSMA targeted surgery, all dissected specimens were sent to pathology for histopathological examination. Specimens were formalin-fixed, sliced into 1–2 mm segments, and paraffin-embedded, after which 3 μm thick sections were obtained and stained with H&E, and if needed immunohistochemical staining for pancytokeratin AE1/3 (CK AE1/3). Disease positivity was defined as the presence of any PCa deposit in the examined specimens. Pathological assessment was performed by experienced uropathologists.

### Statistics

Descriptive statistics are presented as medians and interquartile range (IQR) or as frequencies with percentages. Statistical tests were performed using Python 3.11.12.

## Results

### In vivo target identification of SNs

**Fig. 4:**
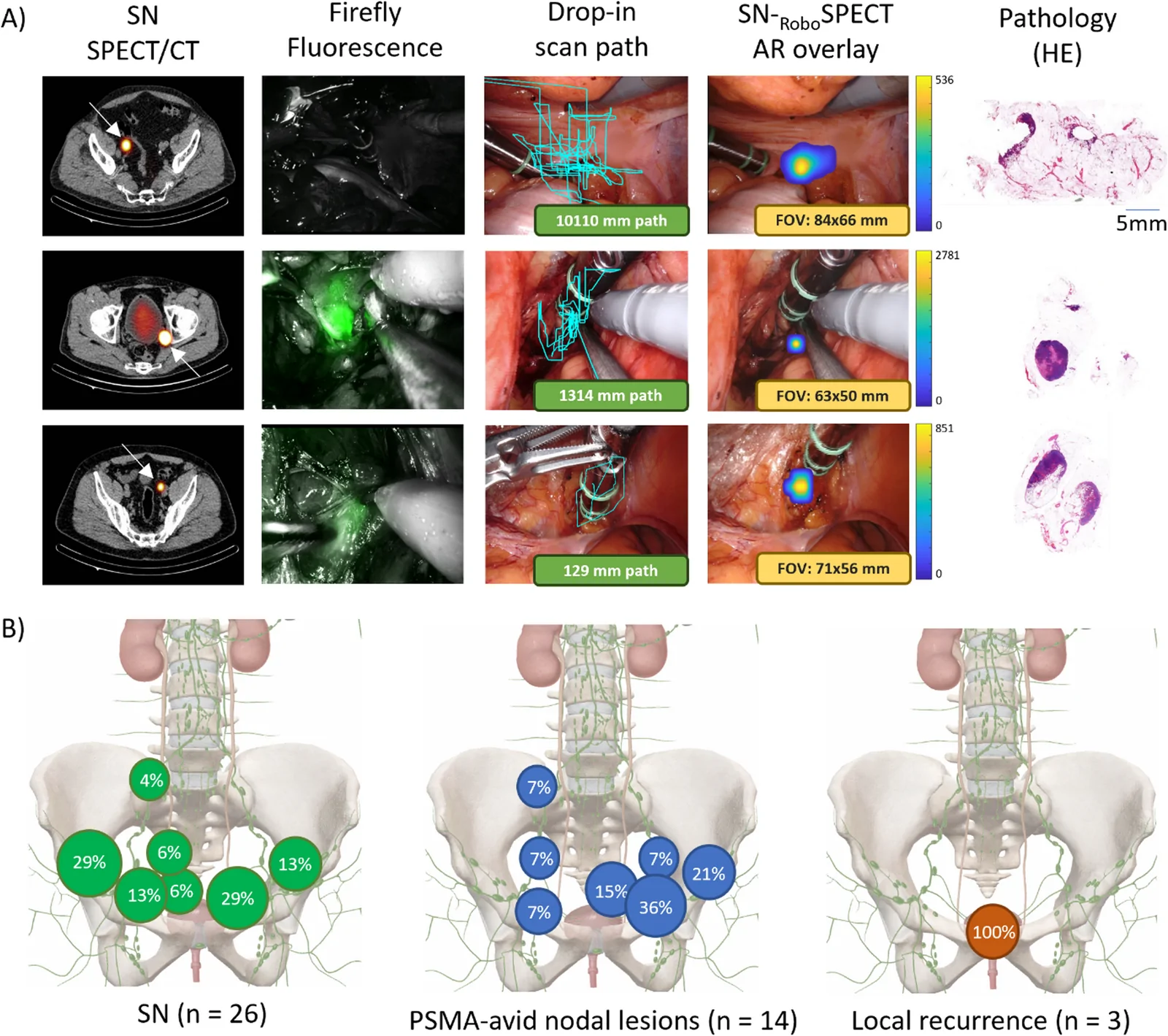
Pre- and intraoperative findings of the 3 tumor positive SNs identified in the study. (**A**) From left to right. First, the anatomical SN location on preoperative SN-SPECT/CT (white arrows). Second, the matching intraoperative Firefly fluorescence images of these SNs with signal in green (top: fluorescence not visible, middle: fluorescence clearly visible, and bottom: fluorescence faintly visible). Third, the intraoperatively tracked drop-in scanning path (blue line overlayed on the white light endoscopic view) and its length in mm. Fourth, the augmented reality (AR) overlay SN-_Robo_SPECT images. Fifth, the nodal tumor content as defined at pathology via HE staining and a scale bar for reference. (**B**) Location and relative distribution of “hot” lesions within the pelvic anatomy

Prior to surgery SPECT/CT roadmaps indicated a total of 28 SNs (10 patients; median 3 SNs per patient, IQR 23). These SNs resided in various anatomical locations including, common, external and internal iliac artery, (deep) obturator fossa, presacral and paravesical (Fig. 4B); Common iliac L (4%), paravesical R (6%), presacral R (6%), external iliac L (13%), external iliac R (29%), obturator fossa R (13%), obturator fossa (29%).

Of the 28 SNs, 5 SNs that were seen on SPECT/CT were not removed because they were in surgically difficult to reach anatomical locations prone to surgical complications (mesorectal, perirectal, and presacral). The remaining 23 SNs were identified using the drop-in gamma sensor (median counts: 322, IQR 140–749; median SBR: 7.5, IQR 4–14) and could be confirmed via ex vivo examination (Table 1). An additional 3 SNs were identified with the drop-in gamma sensor and removed that were not seen on SPECT/CT (total 26 SNs). Moreover, during the surgery 7 additional tissue samples (4 non-SNs and 3 non-lymphatic tissue lesions) were removed at the discretion of the surgeon. All 3 tumor positive lesions were SNs (false negatives 0%; diameter 0.8–5 mm). These cases are depicted in Fig. 4A. For superficial SNs (19/26 SNs (73%)), topological concordance could be achieved between SN-_Robo_SPECT and endoscopic fluorescence imaging (see Fig. 4A; Table 2). In line with previous work [12], the inability to identify deeper lesions with fluorescence underlines the added value provided by SN-_Robo_SPECT. Using the latter accurate visualization was provided for all 26 SNs (sensitivity = 100%) (see Table 2).

**Table 2:** Intra- and post-operative diagnostic data

Surgical procedure (tracer/patients)	PSMA-PET/CT	SPECT/CT	Intraoperative fluorescence	Drop-in sensor counts/s (SBR)	_Robo_SPECT	Tumor positive at PA	Sensitivity	Positive margins after surgery	BCR (at 6 months)
SN (ICG-^99m^Tc-nanocolloid/*n* = 10)	0 lymph nodes	28 lesions (‘reference image’)	19 lesions (73%)	Median 322 (median 8)	26 lesions (5 left in situ; 3 additional)	3 nodes	100%	n.a.	0% in 6 patients
PSMA (99mTc-PSMA I&S/*n* = 11)	14 recurrences (‘reference image’)	4 lesions (29%)	n.a.	Median 19 (median 3)	14 lesions (1 not pursued, 1 positive margin)	14 nodes, 2 local recurrences, 1 positive margin	78%	0 (1 removed at surgery)	20% in 10 patients

*PA* pathology,  *BCR* biochemical recurrence, *SBR* signal-to-background ratio

Figure 4A Illustrates that the scan path was longest (104 mm) for the tumor positive SN that the surgeon could not detect via fluorescence imaging as it was too deep.

### In vivo target identification of PSMA positive lesions

**Fig. 5:**
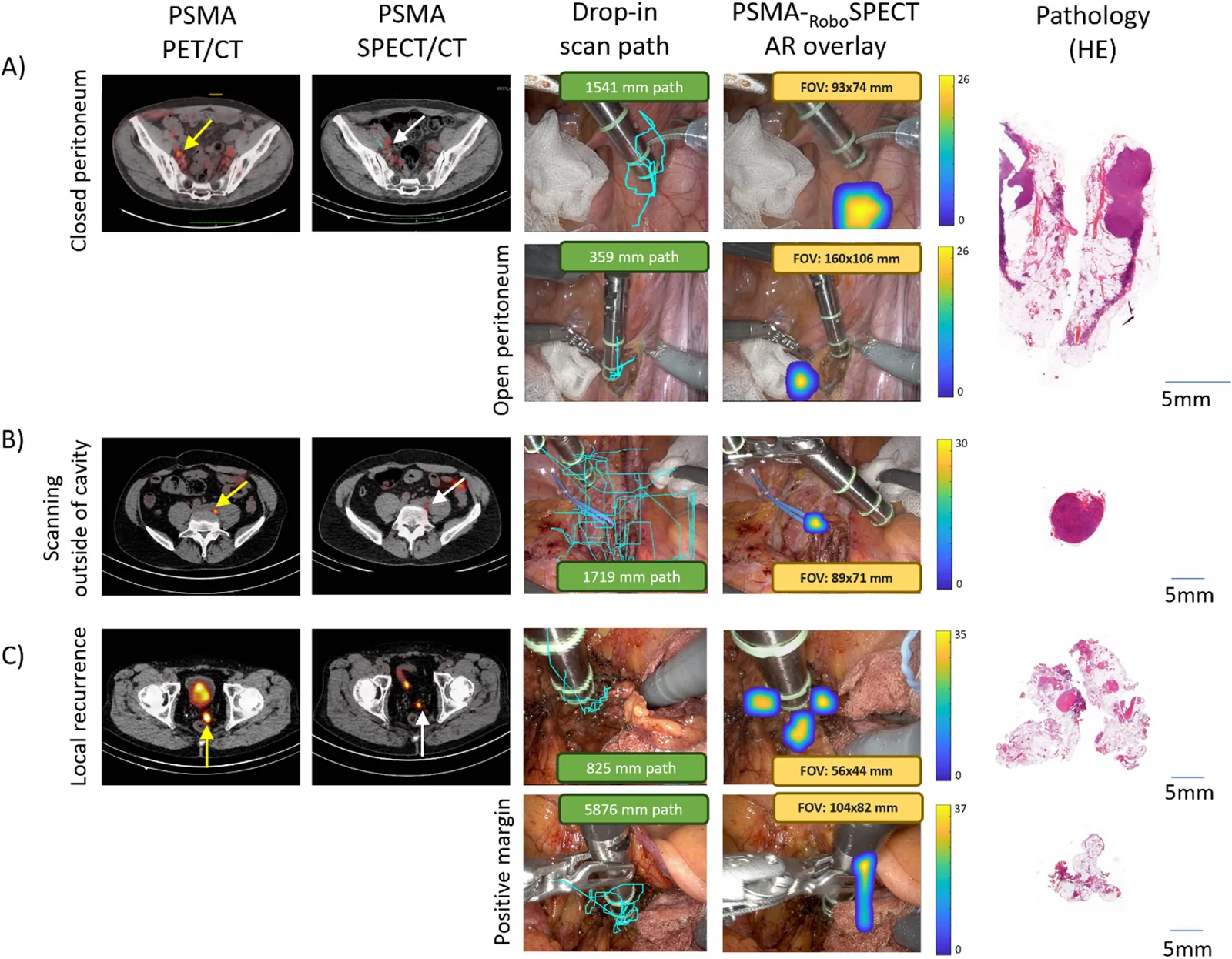
Variations in PSMA-_Robo_SPECT findings**.** From left to right. First, PSMA-SPET/CT (yellow arrows indicate lesion locations). Second, PSMA-SPECT/CT (white arrows indicate lesion locations). Third, the intraoperatively tracked drop-in scanning path (blue line overlayed on the white light endoscopic view) and its length in mm. Fourth, the augmented reality (AR) overlay PSMA-_Robo_SPECT images. Fifth, the nodal tumor content as defined at pathology via HE-staining and a scale bar for reference. From top to bottom images of 3 different patients. (**A**) Patient example 1: Differences in scanning and PSMA-_Robo_SPECT before and after creating the pseudoperitoneum of the same nodal target. (**B**) Patient example 2: Scanning paths are longer when scanning is performed outside of the dedicated wound opening. (**C**) Patient example 3: Use of PSMA-_Robo_SPECT to identify a local recurrence and the positive margins that followed the initial resection

Diagnostic PSMA PET/CT indicated 14 lesions in 10 patients, of which only one was false positive compared to pathology; In one of the patients, we could not properly synchronize the endoscopic and gamma readouts, so that he had to be excluded from further analysis. PSMA-SPECT/CT was only able to visualize 4 lesions in 3 patients (22% true positive lesions). One of the targets identified at PSMA-PET/CT was not pursued with the drop-in sensor. A total of 17 ^99m^Tc-PSMA-avid “hot” lesions (median counts: 19, IQR 13–31; median SBR: 3, IQR 2–3) were surgically targeted: 14 nodal recurrence (2 cluster nodes) and 2 local recurrence lesions and 1 positive resection margin. PSMA-_Robo_SPECT imaging identified all 13 PSMA-PET/CT-positive lesions (100%) as well as the 1 positive resection margin (for examples see Fig. 5).

Targets were distributed over diverse anatomical locations (i.e., common, external and internal iliac arteries, (deep) obturator fossa, pararectal, paravesical, vas deference, seminal vesical; Fig. 4B); Common iliac L (7%), internal iliac L (7%), internal iliac R (7%), pararectal (15%), external iliac L (21%), obturator fossa R (7%), obturator fossa L (36%), and local recurrence (vas def L (33%), Vesicle (top) L (33%), surgical margin rectum (33%). At pathology, tumor was identified in 18 lesions: 15 nodal (diameter 3–23 mm), 2 local recurrence lesions (diameter 2–7 mm), and 1 positive margin (diameter 2 mm). 30 lesions that were not considered “hot” were additionally removed as part of the one-sided lymph node dissection (total 47 lesions), and only 2 (diameter 3 and 4 mm) of these contained tumor (Tables 1 and 2). The drop-in sensor surgically identified two tumor positive (cluster) lesions, for which it was not possible to reconstruct PSMA-_Robo_SPECT images due to insufficient tracking data. As a result, the overall sensitivity of PSMA-_Robo_SPECT and PSMA-PET/CT in relation to pathology were 78% and slightly lower than the sensitivity of the parental drop-in tracing (89%).

PSMA-_Robo_SPECT could be repeated during the procedure, allowing scans to be made before and after dissecting different anatomical planes. Doing so helped to illustrate how spatial constraints influenced the tracing of the targets. For example, the acquisition (volume) was of direct influence on the scanning path. The example in Fig. 5A shows a 4-fold reduction in the scanning path length for the same lesion. Whereby the scanning path length was substantially longer when the scan was made outside of the peritoneum. Interestingly, during the resections of local recurrences, PSMA-_Robo_SPECT was also able to identify a positive resection margin that occurred following the guided resection of the local recurrence (Fig. 5C). Hereby the scanning pathlength required to accurately identify the positive resection margin proved to be 7-fold longer than that of the local recurrence.

## Discussion

This study demonstrates the feasibility, safety profile and alignment with reference modalities of the novel tomographic image-guided surgery modality _Robo_SPECT. By translating _Robo_SPECT to the operating room, we have put forward proof-of-concept data for a unique new gantry-free imaging strategy that supports in-depth molecular (tumor-)target delineation during robotic endoscopic surgery.

One of the benefits of radioguided surgery is that intraoperative findings can be related to preoperative reference scans [4]. In the included SN procedures – where a hybrid tracer was used – _Robo_SPECT visualizations identified 100% of the targets seen on preoperative SN-SPECT/CT. Thereby outperforming fluorescence imaging of the same hybrid tracer (mere 73% visualization rate; see Table 2; Fig. 4A). PSMA-_Robo_SPECT also fully aligned with diagnostic PSMA-PET/CT (100%), thereby substantially improving the mere 29% visualization rate of preoperative PSMA-SPECT/CT (see Table 2). A finding that aligns with various PSMA-targeted image guided surgery studies that underscore that PSMA-SPECT/CT visualization rates are inferior to PSMA-PET/CT [7, 30, 31]. Because PSMA-PET/CT scans remain available for surgical reference, one could argue that recent advances in intraoperative radioguidance could effectively make the PSMA-SPECT/CT scans obsolete. Especially when one realizes that omitting the PSMA-SPECT/CT would improve the workflow (PSMA-SPECT/CT equals a 15–20 min scan duration with image interpretation of a nuclear medicine physician on top), reduce healthcare costs, and minimize the patient burden.

Following its initial clinical translation the drop-in gamma sensor has transformed robotic radioguided surgery [32]. While drop-in scanning of high-count regions helps surgeons identify the position on the tissue surface beyond which a radiopharmaceutical-avid target is located, essentially the technique is non-dimensional (0D). Meaning there is a lack of feedback in terms of the depth the target resides. Conversion of drop-in scans to _Robo_SPECT images (about 3 min reconstruction time) provides insight in position of the lesion below the dissection plane (see distance indications Figs. 3, 4 and 5). Something that is especially valuable when fluorescence cannot act as positional-reference for superficial nodes (Figs. 2 and 27% of SN cases in this study) and/or is not available as reference (all PSMA-targeted procedures). Unfortunately, in contrast to phantom studies [23], quantification of the 3D spatial accuracy is impaired by the tissue deformations that constantly occur when the instruments move. Hence, we have relied on in vivo gamma and fluorescence detection, ex vivo validation measurements, pathological examination of tissue samples, and BCR follow up as references for all targets (see Table 2).

It is commonly assumed that visuals help overcome numeracy and improve understanding. The reported value of mobile gamma cameras over gamma probes when applied in open surgery [33] and the popularity of fluorescence imaging among surgeons suggests this is also true for image guided surgery. By complementing the numerical drop-in sensor readout with a _Robo_SPECT image we have been working towards refining the (visual) feedback to the surgical end-user [34]. The drop-in sensor third-person tracing of high-count regions and _Robo_SPECT first person imaging of these targets tailor robotic interactive perception [35], meaning that during the execution of intraoperative imaging, every motion of the robot is instigated by a surgeon-robot-patient interaction and the surgeon’s decision-making skills [36]. Thus allowing the experienced surgeons involved in this study to adapt their scans to unique scene changes and identify targets in a range of surgical scenarios, most of which were inherently unpredictable due to the anatomical variations between patients. _Robo_SPECT could visualize a range of ^99m^Tc- avid targets: SNs, PSMA-avid LNs, local recurrences and a positive margin. This could be realized despite the variations in: scan durations (IQR 19–48 s), signal intensities (Median range 19–322; See Table 2) and signal to background ratios (Median range 3–8; See Table 2). Our observations do, however, suggest that scan paths were shorter (more focused) when surgeons were confident of the lesion location e.g., due to fluorescence-based confirmation (see Fig. 4A), while at the same time lengthening when accessibility of the target allowed (see Fig. 5A open vs. closed peritoneum). Features that warrant further investigation in future studies.

_Robo_SPECT images in this study were based on conventional drop-in scans made at the surgeon’s discretion. Unfortunately, not every scan could be converted into an image. Effects related to tracking inaccuracies impaired _Robo_SPECT image reconstruction of 2 tumor positive PSMA lesions, negatively reflecting on the pathology related sensitivity. Thus, indicating that there is room to further improve the tracking aspect behind _Robo_SPECT. Sensor tracking is currently reliant on the unique geometry of the second-generation CE-marked drop-in gamma-sensor housing and the three PEEK fiducials (see Fig. 2). Literature describes the use of alternative barcode-like stickers for drop-in sensor tracking in patients [37, 38]. Furthermore, marker-less artificial intelligence drop-in sensor tracking is clinically feasible [7]. Both of which can be explored to help refine the drop-in kinematic tracking-architecture in the future.

Holding the future of surgery in mind, it is evident that – in analogy to self-driving cars – embedded quantitative and in-depth sensory readouts (See Figs. 4 and 5) [7] help provide the technological thrust needed to pursue increased autonomy of robotic surgical systems [39]. By firmly establishing a link between precision surgical decision making and intraoperative molecular imaging, the latter field seems to become increasingly more important in the future of surgical care.

## Conclusions

We have successfully extended non-dimensional numerical drop-in gamma tracing, with augmented reality displays of _Robo_SPECT images that provide 3D insights in the lesion location. _Robo_SPECT showed a favorable correlation to pathology and outcome measures, while proving reproducible under a wide range of conditions. In the SN-group _Robo_SPECT clearly outperformed fluorescence imaging, while in the PSMA- group it outperformed preoperative PSMA-SPECT/CT imaging.

## Data Availability

Data can be made available upon reasonable request, when it does not serve commercial interests and under a confidentiality clause.

## References

[1] Beasley RA. Medical robots: current systems and research directions. Journal of Robotics. 2012. .

[2] Moustris GP, et al. Evolution of autonomous and semi-autonomous robotic surgical systems: a review of the literature. Int J Med Robot. 2011;7(4):375–92. 10.1002/rcs.408 21815238

[3] Mazzone E, et al. Diagnostic value, oncological outcomes and safety profile of image-guided surgery technologies during robot-assisted lymph node dissection with sentinel node biopsy for prostate cancer. J Nucl Med. 2021. . 10.2967/jnumed.120.259788 PMC8724888 33547208

[4] van Leeuwen FWB, et al. The rise of molecular image–guided robotic surgery. J Nucl Med. 2024;65(10):1505. 10.2967/jnumed.124.267783 38991755

[5] Gandaglia G, et al. Prostate-specific membrane antigen radioguided surgery to detect nodal metastases in primary prostate cancer patients undergoing robot-assisted radical prostatectomy and extended pelvic lymph node dissection: results of a planned interim analysis of a prospective phase 2 study. Eur Urol. 2022;82(4):411–8. 10.1016/j.eururo.2022.06.002 35879127

[6] Maurer T, et al. PSMA-PET for lymph node detection in recurrent prostate cancer: how do we use the magic bullet? Theranostics. 2017;7(7):2046–7. 10.7150/thno.20855 PMC5479286 28638485

[7] Azargoshasb S, et al. Artificial intelligence-supported video analysis as a means to assess the impact of DROP-IN image guidance on robotic surgeons: radioguided sentinel lymph node versus PSMA-targeted prostate cancer surgery. Adv Intell Syst. 2023;5(10):2300192.

[8] Vermeeren L, et al. Value of SPECT/CT for detection and anatomic localization of sentinel lymph nodes before laparoscopic sentinel node lymphadenectomy in prostate carcinoma. J Nucl Med. 2009;50(6):865–70. 10.2967/jnumed.108.060673 19443579

[9] Dell’Oglio P, et al. A DROP-IN gamma probe for robot-assisted radioguided surgery of lymph nodes during radical prostatectomy. Eur Urol. 2021;79(1):124–32. 10.1016/j.eururo.2020.10.031 33203549

[10] Berrens A. Strong correlation between SUVmax on PSMA PET/CT and numeric drop-in γ-probe signal for intraoperative identification of prostate cancer lesions. J Nucl Med. 2024. . 10.2967/jnumed.123.267075 38485277

[11] Brouwer OR, et al. Feasibility of intraoperative navigation to the sentinel node in the groin using preoperatively acquired single photon emission computerized tomography data: transferring functional imaging to the operating room. J Urol. 2014;192(6):1810–6. 10.1016/j.juro.2014.03.127 25066868

[12] Meershoek P, et al. Can intraoperative fluorescence imaging identify all lesions while the road map created by preoperative nuclear imaging is masked? J Nucl Med. 2020;61(6):834–41. 10.2967/jnumed.119.235234 31712325

[13] van der Poel HG, et al. Intraoperative laparoscopic fluorescence guidance to the sentinel lymph node in prostate cancer patients: clinical proof of concept of an integrated functional imaging approach using a multimodal tracer. Eur Urol. 2011;60(4):826–33. 10.1016/j.eururo.2011.03.024 21458154

[14] Oosterom MNv, et al. Extending the hybrid surgical guidance concept with freehand fluorescence tomography. IEEE Trans Med Imaging. 2020;39(1):226–35. 10.1109/TMI.2019.2924254 31247546

[15] Knipper S, et al. Biochemical response of < 0.1 ng/ml predicts therapy-free survival of prostate cancer patients following prostate-specific membrane antigen-targeted salvage surgery. Eur Urol Oncol. 2025;8(2):270–7. 10.1016/j.euo.2024.04.019 38729805

[16] Berrens AC, et al. State of the art in prostate-specific membrane antigen-targeted surgery-a systematic review. Eur Urol Open Sci. 2023;54:43–55. 10.1016/j.euros.2023.05.014 PMC10285550 37361200

[17] Berrens A-C, et al. Delphi consensus project on prostate-specific membrane antigen (PSMA)–targeted surgery—outcomes from an international multidisciplinary panel. Eur J Nucl Med Mol Imaging. 2024;51(10):2893–902. 10.1007/s00259-023-06524-6 38012448

[18] Wendler T, et al. Towards intra-operative 3D nuclear imaging: reconstruction of 3D radioactive distributions using tracked gamma probes. Med Image Comput Comput Assist Interv. 2007;10(Pt 2):909–17. 10.1007/978-3-540-75759-7_110 18044655

[19] Bluemel C, et al. 3D scintigraphic imaging and navigation in radioguided surgery: freehand SPECT technology and its clinical applications. Expert Rev Med Devices. 2016;13(4):339–51. 10.1586/17434440.2016.1154456 26878667

[20] Schilling C, et al. Intraoperative sentinel node imaging versus SPECT/CT in oral cancer – A blinded comparison. Eur J Surg Oncol. 2018;44(12):1901–7. 10.1016/j.ejso.2018.08.026 30236825

[21] van Matthias Nathanaël O, et al. Navigation of a robot-integrated fluorescence laparoscope in preoperative SPECT/CT and intraoperative freehand SPECT imaging data: a phantom study. J Biomed Opt. 2016;21(8):086008. 10.1117/1.JBO.21.8.086008 27548770

[22] Azargoshasb S et al. Optical navigation of a DROP-IN gamma probe as a means to strengthen the connection between robot-assisted and radioguided surgery. J Nucl Med. 2021:p. jnumed.120.259796. 10.2967/jnumed.120.259796 PMC8882900 33419942

[23] Fuerst B, et al. First robotic SPECT for minimally invasive sentinel lymph node mapping. IEEE Trans Med Imaging. 2016;35(3):830–8. 10.1109/TMI.2015.2498125 26561283

[24] Azargoshasb S, et al. Robot-assisted single photon emission computed tomography: integrating nuclear medicine in robotic urologic surgery. Eur Urol. 2024;85(5):503–5. 10.1016/j.eururo.2024.01.022 38369422

[25] Azargoshasb S, et al. The Click-On gamma probe, a second-generation tethered robotic gamma probe that improves dexterity and surgical decision-making. Eur J Nucl Med Mol Imaging. 2021;48(13):4142–51. 10.1007/s00259-021-05387-z PMC8566398 34031721

[26] Alexander H et al. Freehand SPECT reconstructions using look up tables. in Proc.SPIE. 2012.

[27] Hartl A, et al. Detection models for freehand SPECT reconstruction. Phys Med Biol. 2015;60(3):1031. 10.1088/0031-9155/60/3/1031 25585618

[28] Matthies P, et al. Mini gamma cameras for intra-operative nuclear tomographic reconstruction. Med Image Anal. 2014;18(8):1329–36. 10.1016/j.media.2014.04.009 24853626

[29] Shepp LA, Vardi Y. Maximum likelihood reconstruction for emission tomography. IEEE Trans Med Imaging. 1982;1(2):113–22. 10.1109/TMI.1982.4307558 18238264

[30] García-Pérez FO, et al. Head to head comparison performance of (99m)Tc-EDDA/HYNIC-iPSMA SPECT/CT and (68)Ga-PSMA-11 PET/CT a prospective study in biochemical recurrence prostate cancer patients. Am J Nucl Med Mol Imaging. 2018;8(5):332–40. PMC6261875 30510850

[31] Lawal IO, et al. Diagnostic sensitivity of Tc-99m HYNIC PSMA SPECT/CT in prostate carcinoma: a comparative analysis with Ga-68 PSMA PET/CT. Prostate. 2017;77(11):1205–12. 10.1002/pros.23379 28649735

[32] Pisano G, et al. Flexible intraoperative detectors for robotic and laparoscopic image-guided surgery: a systematic review. Eur J Nucl Med Mol Imaging. 2026. 10.1007/s00259-025-07715-z 41665674

[33] Heller S, Zanzonico. Nuclear probes and intraoperative gamma cameras. Semin Nucl Med. 2011;41(3):166–81. 10.1053/j.semnuclmed.2010.12.004 21440694

[34] van Erp C et al. High fidelity intraoperative visualization of primary prostate cancer in 3D. 2025, Leiden University Medical Centre: EJNMMI.

[35] Bohg J, et al. Interactive perception: leveraging action in perception and perception in action. IEEE Trans Robot. 2017;33(6):1273–91.

[36] Marinho MM et al. A unified framework for the teleoperation of surgical robots in constrained workspaces. in 2019 International Conference on Robotics and Automation (ICRA). 2019.

[37] Ma L, et al. Robust and fast laparoscopic vision-based ultrasound probe tracking using a binary dot array marker. Comput Biol Med. 2022;145:105406. 10.1016/j.compbiomed.2022.105406 35339847

[38] Hughes-Hallett A, et al. Intraoperative ultrasound overlay in robot-assisted partial nephrectomy: first clinical experience. Eur Urol. 2014;65(3):671–2. 10.1016/j.eururo.2013.11.001 24262103

[39] Thai MT, et al. Advanced intelligent systems for surgical robotics. Adv Intell Syst. 2020;2(8):1900138.

